# Mortality and morbidity burden associated with A/H1N1pdm influenza virus: Who is likely to be infected, experience clinical symptoms, or die from the H1N1pdm 2009 pandemic virus ?

**DOI:** 10.1371/currents.RRN1013

**Published:** 2009-09-02

**Authors:** Mark Miller, Cecile Viboud, Lone Simonsen, Donald R. Olson, Colin Russell

**Affiliations:** ^*^National Institutes of Health; ^†^Fogarty International Center, National Institutes of Health, Bethesda, MD, USA; ^‡^Department of Global Health, George Washington University, School of Public Health and Health Services, DC; ^§^International Society for Disease Surveillace and ^¶^University of Cambridge & Fogarty International Center - NIH

## Abstract

Here we use lessons from past influenza pandemics and recent information about the H1N1pdm pandemic to discuss variations in H1N1pdm disease burden with age, underlying risk factors, and geography.

Influenza is predominantly caused by influenza viruses type A and B which continuously circulate amongst humans throughout the world.  In temperate climates, influenza seasons occur typically during the late fall to late winter with peak periods during the winter.  In tropical and sub-tropical regions, influenza viruses circulate year round with less seasonal activity. [1] [2] [3]

## Influenza viruses during epidemics and  pandemics

Type A influenza viruses are further classified by sub-type based on the viral surface antigens, the hemaglutinin (HA) and neuraminidase (NA). Antibodies to the HA are considered protective against infection, while those against NA reduce disease severity.  There are 16 known sub-types of HA circulating in birds, the natural reservoir of influenza viruses; only three, thus far, are known to result in **continuous** transmission amongst humans (viruses containing hemaglutinin subtype 1 A(H1), subtype 2 A(H2) and subtype 3 A(H3)).   (While Type B viruses also infect humans, illnesses are typically less severe and none are known to have caused true pandemics).  The eight gene segments of the different viruses which infect birds and mammals can reassort and sometimes create viruses with a new HA, which can transmit among humans, who have very little immunity -- resulting in a **pandemic**.  Over the last 91 years, four of these reassortant or HA **shift** in viruses have resulted in successful human-to-human transmission and caused pandemics (A(H1) in 1918, A(H2) in 1957, A(H3) in 1968, A(H1pdm) in 2009. In the 1957 and 1968 pandemics, the novel influenza virus replaced the previously circulating subtype (this also presumably occurred in 1918 based on antigenic studies, though it cannot be stated definitively as there has been no human influenza virus isolated from before 1918).  In 1977 when A(H1) viruses re-emerged virtually unchanged after a 20-year absence, they did not become dominant, as measured by the majority of isolates recovered from ill individuals [4], but instead co-circulated with the A(H3) viruses.    

The emerging A(H1pdm) virus of  2009 may or may not replace the previously circulating A(H1) and A(H3) viruses. As of Aug 2009, seasonal A(H1) and A(H3) viruses continue to circulate along with A(H1pdm), although generally at lower levels than usual [5].

### Prior immunity to influenza viruses in 2009

For almost all persons born from 1918 to 1957 (~52 to 91 year olds in 2009), the first exposure to an influenza A virus was to the strains containing A(H1); those born from 1957 to 1968 (~41 to ~52) to  A(H2); those born since the last pandemic in 1968 (<41 years of age), most likely to A(H3).  Indeed, the A(H1) subtype has been reintroduced in 1977 but is rarely dominant [5],  suggesting that most people born after 1977 were first exposed to A(H3) viruses. This is important because the concept of “original antigenic sin” postulates that the virus sub-type that one is first exposed to in childhood will provide the greatest immunity in later years [6] [7].  Therefore, those persons born prior to 1957 may have the greatest natural immunity to the currently circulating  A(H1pdm) pandemic virus  in 2009 [8]. 

These age groups provide a rough guide as to who was infected with each virus sub-type as not all individuals may have been infected in childhood at the time of emergence of pandemic viruses.   

### Influenza infection and transmission

Influenza viruses are highly transmissible person to person through the respiratory route with a reproduction number ranging between 1.1 and 1.8 and a serial interval of 2-3 days  during seasonal epidemics, meaning that each case infects 1.1 to 1.8 other individuals every 2-3 days on average[9].  Transmission amongst individuals is believed to vary with multiple factors including age, population density, contact rates, climatological conditions and immunity profiles within populations.  Children have the highest contact rates and can accelerate local transmission, while adults are known to spread the virus over greater distances [10] [11] [12]. An estimated 5 to 20% of the world’s population become infected during each seasonal epidemic [13].  It has been reported that up to 50% of Infants with siblings become infected during an influenza season.[14]

During pandemic seasons, as a greater proportion of the population is susceptible, transmission is higher, with an estimated R=1.5-5.5 secondary cases [15] [16] [17] [18] [19] [20] [21] especially in younger populations, and a larger fraction of the population experiences clinical symptoms, estimated at around 30% [22] [23].  One study documented 85% of children to be infected in Cleveland during the first year of the 1957 pandemic [22]. 

The new sub-type virus may circulate for several years although it will mutate or will genetically **drift** over that time and continue to cause epidemics until replaced with a new pandemic virus. Hence, with each new shift of a new influenza virus, with a high initial year attack rate and an annual seasonal attack rate on the order of 5 to 20% everyone will eventually become infected with the new pandemic strain over several years. This may occur in several waves [24].

While most influenza epidemiology studies were set in developed countries, evidence from the Gambia shows over 90% seroconversion with multiple infections of the A(H3) pandemic virus within 4 years of the 1968 pandemic.[25] 

### Signature Features of Pandemics

As older individuals may have been previously exposed to previously circulating influenza virus subtypes during their childhood, the mortality and morbidity burden of a pandemic shifts to a younger population with most infections and mortality occurring during the earliest phases of the pandemic [26] [27]. Aside from the shift of the dominant circulating viral sub-type and in the age patterns of deaths and illnesses,  rapid transmission due to the greater susceptibility in the general population, there are other signature features of pandemics, including multiple waves of morbidity and mortality over the first few months or years, and disruption from the typical winter seasonality observed in seasonal epidemics [24]. There is also great variation of pandemic health burden amongst populations [28],  although the impact of influenza remains poorly studied in developing country populations. In the most severe reported 1918-20 pandemic, mortality was estimated to range from 0.2% in Scandinavian countries to nearly 8% in some regions in India [28], a difference  of ~40 fold.  Of note, there may be issues of ascertainment bias from historical data, and these mortality rates are not directly applicable to the current H1N1pdm outbreak as circulating viruses, populations, and treatment capabilities have dramatically changed since 1918.

### Illness and death from seasonal and pandemic influenza

While it is expected that 100% of the population to be eventually infected with the new pandemic virus, the virulence and pathogenicity of the virus is related to multiple factors, many of which are somewhat independent of infectivity.  While influenza viruses may be highly infectious, they may not necessarily cause much disease burden in the general population. 

Other viral factors may contribute to severity of influenza-related infection, including virulence factors in HA, NS1 and PB1[29] [30] [31].   Virulence is also dependent on various host factors; some well established or hypothesized such as co-morbid conditions, obesity, pregnancy, pneuomococcal carriage, host genomics, and perhaps protection from prior exposure to sub-type virus in childhood  (i.e. those born prior to 1957).  

## Relevance to the current H1N1pdm pandemic 

### Impact of the first A/H1N1pdm pandemic wave, Apr-Aug 2009.

Since April 2009, most countries have reported laboratory-confirmed cases of H1N1pdm.  It is also clear that these counts would greatly underestimate the total burden of the first pandemic wave, as most cases would not have been confirmed.  For example, CDC had discouraged physicians from laboratory testing due to overwhelmed laboratory system a few weeks into the outbreak.  Consequently, the count of ~100K cases in late July was known to be severely undercounted; instead, CDC estimated that over 1M persons had been infected in the US.  

The 1^st^ wave of H1N1pdm in summer 2009 can be characterized as mild, relative to the mortality impact of severe winter-seasonal influenza epidemics.  However the H1N1pdm outbreak is associated with the “signature age shift” that is typical for influenza pandmics.  Morbidity and mortality have tended to occur in adults aged 20-50 years [8], with some deaths in children, and largely sparing of seniors.  Both of these features – a mild summer wave with elevated mortality in young adults resembles the pattern of the 1918 pandemic, in which a first spring wave in the US  [32] or summer wave in Europe [33] was associated with unusual deaths in young adults.  The subsequent catastrophic wave in autumn 1918 killed about 2% of the population world-wide, with a dramatic mortality elevation in young adults [28]. Of note, the 1^st^ wave of the H1N1pdm virus has had a very different morbidity impact in various US cities and regions, something that cannot easily be explained by contemporary mathematical models.  For example, about 7% of persons living in NY City are thought to have been infected this summer, while other cities did not see much of an elevation in influenza illness [34].  Similarly, while Argentina has been experiencing an emergency situation, surrounding countries like New Zealand have had far less of an impact [35].  Such variability of impact have previously been noted for 1918-20 pandemic  and attributed to differences in access to care and overall risk of dying in developing countries [27].

The early phases of recognition of emerging infections almost always show a high case fatality rate due to the dramatic ascertainment of cases and the unknown extent of infections that have already occurred.   Media and public health alarm bells are frequently sounded until there is a clearer understanding of the degree of background infections are measured as well as transmission rates.  Limited laboratory diagnostic tests and uncertain definitions of disease during the early phase lead to frequent misunderstandings. With improved sensitive and specific diagnostics and population based surveys, a clearer picture emerges.    

The uncertainly about the number of people infected (=denominator for case fatality rate) is critical when it comes to ascertaining the case fatality ratio and thus getting a stronger sense of the severity of this pandemic.  It is extremely difficult to gauge how many people have been infected thus far, based on passive surveillance data available, which consists mostly of deaths, hospitalizations, or influenza-like-illnesses. The proportion of symptomatic cases who seek care and are captured by existing surveillance systems remains unclear is is bound to vary across countries and settings, and with media addition as the pandemic progresses. So far, estimates of the case fatality rate range between 1 per 10,000 for Acute Respiratory Distress Syndrom (see [36]) to 1 per 2,000 for all influenza-related deaths. These estimates come with large cofnidence intervals and are likely to change over time. Of note, the case fatality rate for seasonal influenza is 1 per 1,000 on average, which appears more severe than H1N1pdm, however one has to remembers that it is mostly driven by a high proportion of deaths among seniors. By contrast, H1N1pdm-related deaths are concentrated in people under 60 yrs, a population typically at low risk of influenza-related deaths.

In addition, an important quantity to gauge disease severity is the proportion of asymptomatic cases, which remains largely unknown (for H1N1pdm as well as for seasonal influenza viruses). The proportion of asymptomatic cases can only be measured by costly prospective studies combining serological outcomes to measure evidence of infection, with a clinical follow-up of patients to monitor symptoms. Such studies were conducted in the 1940-1970s in US and British households and proved  instrumental in monitoring the impact of past pandemics (eg, [22]). So far, there has not been such studies for H1N1pdm, although some may be under way. 

 In the meantime, to monitor trends in H1N1pdm burden over time or across age groups, we can rely on existing passive surveillance systems which are insensitive to the ascertainment issues – for example, CDCs 122 mortality system, which showed only a moderate elevation of mortality during the 1^st^ wave in summer in the US [37].  Thus, at least in the US, it is clear that the 1^st^ wave was not associated with a measurable amount of excess mortality above the seasonal baseline.  This is not to say that severe outcomes and deaths have not occurred; CDCs pediatric surveillance system shows that influenza related hospitalizations and deaths occurred nationwide; however, the numbers were definitely lower than that attributed to seasonal influenza during the last 4 influenza seasons.   

 But some other countries have reported high hospitalization impact.  For example, in the Southern hemisphere where the influenza season is normally during May-September, the impact has been reported to be “the worst in the last decade” in New Zealand and Argentina, with ICUs filled.   But also the UK had an intense first wave, despite high use of antivirals for prophylaxis and treatment.  In Mexico, adults aged 20-50 had a clearly elevated hospitalization rate than for a comparison period in previous years [8].

Clearly, more data are urgently needed to assess the mortality and morbidity burden of the first wave of H1N1pdm, and  quantify variation across countries and age groups.  

### What will happen in the 2nd wave expected this autumn?

Even though the similarities of the epidemiology of H1N1pdm and the 1918 pandemic are concerning, there is no way of knowing whether the current H1N1pdm virus will cause a more severe 2^nd^ wave in the coming fall.  The virus may or may not acquire virulence mutations and become a more pathogenic state in successive waves.  

There are some unique differences in this pandemic from prior pandemics.  As noted earlier, A(H1) viruses have previously circulated in the population, and may have conferred  some level of protection to older age groups, who are relatively spared against morbidity and mortality [8] [38]

 Evolution dictates that success of transmission and ability to compete with other evolving viruses will determine the characteristics of future strains.  There have been historic examples of mild, moderate or severe waves followed in previous pandemics [24]. However, even if this pandemic is at the mild end of scale, it is worth keeping in mind the age shift of severe disease to younger populations, warranting increased attention to populations not normally thought about at greater risk.  

While there is no way to predict the course of this pandemic, careful statistical measurements of age-specific morbidty and mortality data, combined with information on virus activity and genetic characteritics, allows for an assessment of pandemic impact, and is crucial in the first two years of a new virus circulation [39] [26]. 

## Factors that may affect H1N1pdm impact in the waves to come

On the positive side, some factors could attenuate the mortality impact of subsequent waves of H1N1pdm, possibly in the fal, in particular effective pandemic vaccination. In addition, immunity acquired from exposure to H1N1pdm in summer 2009 could protect a fraction of the population against subsequent outbreaks of similar or related viruses in fall [40]. Other factors include antiviral and antibiotic use (to mitigate primary viral infection and secondary bacterial infections), school closings, and effective social mitigation strategies.

On the negative side, other factors could lead to a worsening situation in the coming months.   These include winter-seasonal factors in the Northern Hemisphere, including presence of bacterial pathogens such as S.pneumo and MRSA, who are responsible for a large fraction of influenza-triggered deaths in  seasonal epidemics and past pandemics [41].  As discussed previously, viral acquisition of virulence factors by mutation would be unfortunate, as would acquisition of antiviral resistance determinants.  

## Acknowledgments

We thank Marc Lipsitch and Derek Smith for helpful discussions.

## Funding information

The study was funded by the intra-mural program of the Fogarty International Center, National Institutes of Health.  

## Competing interests

The authors have declared that no competing interests exist.
